# *rac*-1-(6-Hy­droxy-3,6-dimethyl-4-phenyl-4,5,6,7-tetra­hydro-2,1-benzoxazol-5-yl)ethanone

**DOI:** 10.1107/S1600536810043667

**Published:** 2010-10-31

**Authors:** Abel M. Maharramov, Arif I. Ismiyev, Bahruz A. Rashidov

**Affiliations:** a Baku State University, Z. Khalilov Street 23, Baku AZ-1148, Azerbaijan

## Abstract

The structure of the title compound, C_17_H_19_NO_3_, is of inter­est with respect to anti­bacterial properties, anti­biotic properties and biological activity. The structure displays inter­molecular O—H⋯N hydrogen bonding.

## Related literature

For general background to Schiff bases and their uses, see: Lau *et al.* (1999 ▶); Shawali *et al.* (1985 ▶); Raman *et al.* (2003 ▶); Yuxia *et al.* (2002 ▶).
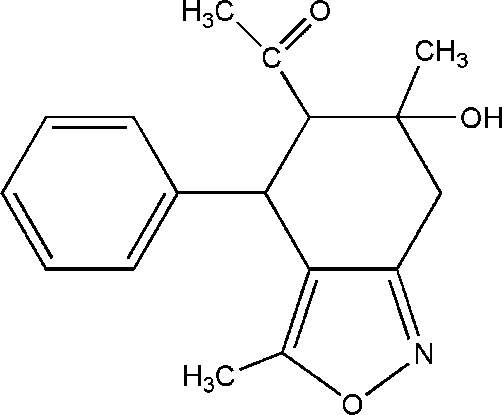


## Experimental

### 

#### Crystal data

C_17_H_19_NO_3_
*M* *_r_* = 285.33
Monoclinic, 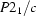

*a* = 16.1518 (9) Å
*b* = 5.5353 (3) Å
*c* = 17.2956 (9) Å
β = 103.496 (1)°
*V* = 1503.61 (14) Å^3^
*Z* = 4
Mo *K*α radiation
μ = 0.09 mm^−1^
*T* = 296 K
0.30 × 0.20 × 0.20 mm

#### Data collection

Bruker APEXII CCD diffractometer
Absorption correction: multi-scan (*SADABS*; Sheldrick, 1998 ▶) *T*_min_ = 0.975, *T*_max_ = 0.983
16629 measured reflections
3724 independent reflections
2840 reflections with *I* > 2σ(*I*)
*R*_int_ = 0.021

#### Refinement

*R*[*F* ^2^ > 2σ(*F* ^2^)] = 0.046
*wR*(*F* ^2^) = 0.141
*S* = 1.00
3724 reflections
190 parameters
H-atom parameters constrained
Δρ_max_ = 0.28 e Å^−3^
Δρ_min_ = −0.21 e Å^−3^

### 

Data collection: *APEX2* (Bruker, 2005 ▶); cell refinement: *SAINT-Plus* (Bruker, 2001 ▶); data reduction: *SAINT-Plus*; program(s) used to solve structure: *SHELXTL* (Sheldrick, 2008 ▶); program(s) used to refine structure: *SHELXTL*; molecular graphics: *SHELXTL*; software used to prepare material for publication: *SHELXTL*.

## Supplementary Material

Crystal structure: contains datablocks global, I. DOI: 10.1107/S1600536810043667/pb2043sup1.cif

Structure factors: contains datablocks I. DOI: 10.1107/S1600536810043667/pb2043Isup2.hkl

Additional supplementary materials:  crystallographic information; 3D view; checkCIF report

## Figures and Tables

**Table 1:** Hydrogen-bond geometry (Å, °)

*D*—H⋯*A*	*D*—H	H⋯*A*	*D*⋯*A*	*D*—H⋯*A*
O3—H3*B*⋯N1^i^	0.82	2.08	2.8689 (17)	162

Symmetry code: (i) 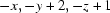.

**Fig. 1.**
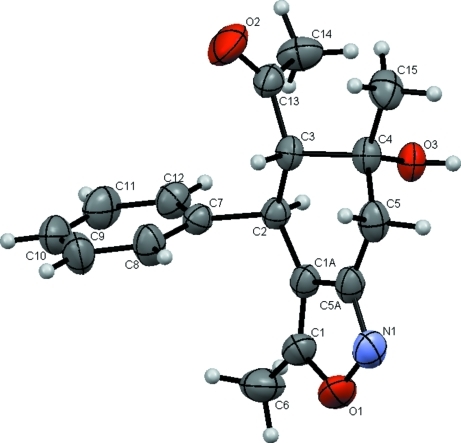
The molecular structure of the title compound, with the atomic numbering scheme.Displacement ellipsoids were drawn at the 30% probability level.

**Fig. 2.**
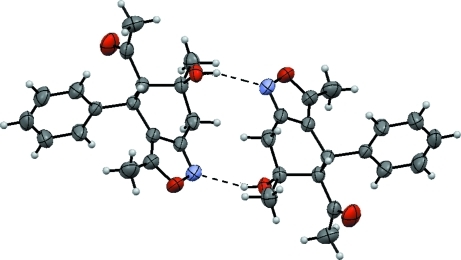
A hydrogen-bonded (dashed lines) ribbon in the title compound. H atoms not involved in hydrogen bonding have been omitted for clarity.

## supplementary crystallographic information

### Comment

Schiff bases are characterized by the –CH═N– (imine) group which is impotantant in elucidating the mechanism of transamination and racemization reactions in biological systems (Lau *et al.*, 1999; Shawali *et al.*, 1985). Due to the great flexibility and diverse structural aspects, a wide range of Schiff bases have been synthesized and their complexation behaviour studied (Raman *et al.*, 2003). Literature survey shows that Schiff bases show bacteriostatic and bactericidal activity (Yuxia *et al.*, 2002). Antibacterial, antifungal, antitumor, anticancer activity has been reported and they are also active against a wide range of organisms. We have synthesized the title compound, (rac)-5-acetyl-6-hydroxy-3,6-dimethyl-4-phenyl-2*H*-4,5,6,7-tetrahydrobenzo[*c*]isoxazole (I), and its structure is reported here. (I) have good antibacterial properties. In the title compound (Fig. 1) all bond lengths and angles are normal. Intermolecular O—H···N hydrogen bonds (Table 1; Fig.2) link molecules into centrosymmetric dimer. The cystal packing is further stablized by van der Waals forces. The two [(C2(*R*),C4(*R*)] of three stereogenic centres of tetrahydrobenzo[*c*]izoxazole moiety are of the same chirality. As the crystal crystallizes in the centrosymmetric space group, the racemate (1:1) is present.

### Experimental

(rac)-2,4-diacetyl-5-hydroxy-5-methyl-3-phenylcyclohexanon (20 mmol), hydroxylamine hydrochloride (20 mmol) were dissolved in 20 ml ethanol. The mixture was stirred at 345–350 K within 10 h. After cooling to a room temperature obtained white crystals. The crystals was filtered and washed with ethanol. Have been then dissolved in ethanol (50 ml) and recrystallized to yield colourless block-shaped crystals of the title compound.The melting point 140 °C.

### Refinement

The H atoms of the OH and NH groups of the molecule (I) were localized in the difference Fourier map and included in the refinement with fixed positional and isotropic displacement parameters [*U*_iso_(H) = 1.5*U*_eq_(C) for CH_3_ group and *U*_iso_(H) = 1.2*U*_eq_(N) for amino groups]. The other H atoms were placed in calculated positions with and refined in the riding model with fixed isotropic displacement parameters [*U*_iso_(H) = 1.2*U*_eq_(C)].)]. 24 reflections, with experimentally observed F^2^ deviating significantly from the theoretically calculated F^2^, were omitted from the refinement. Moreover, 76 reflections were not measured because the angle limits.

### Crystal data

**Table:**

C_17_H_19_NO_3_	*F*(000) = 608
*M**_r_* = 285.33	*D*_x_ = 1.260 Mg m^−^^3^
Monoclinic, *P*2_1_/*c*	Mo *K*α radiation, λ = 0.71073 Å
Hall symbol: -P 2ybc	Cell parameters from 5396 reflections
*a* = 16.1518 (9) Å	θ = 2.4–28.2°
*b* = 5.5353 (3) Å	µ = 0.09 mm^−^^1^
*c* = 17.2956 (9) Å	*T* = 296 K
β = 103.496 (1)°	Prism, colourless
*V* = 1503.61 (14) Å^3^	0.30 × 0.20 × 0.20 mm
*Z* = 4	

### Data collection

**Table:**

Bruker APEXII CCD diffractometer	3724 independent reflections
Radiation source: fine-focus sealed tube	2840 reflections with *I* > 2σ(*I*)
graphite	*R*_int_ = 0.021
φ and ω scans	θ_max_ = 28.3°, θ_min_ = 2.4°
Absorption correction: multi-scan (*SADABS*; Sheldrick, 1998)	*h* = −21→21
*T*_min_ = 0.975, *T*_max_ = 0.983	*k* = −7→7
16629 measured reflections	*l* = −23→22

### Refinement

**Table:**

Refinement on *F*^2^	Primary atom site location: structure-invariant direct methods
Least-squares matrix: full	Secondary atom site location: difference Fourier map
*R*[*F*^2^ > 2σ(*F*^2^)] = 0.046	Hydrogen site location: difference Fourier map
*wR*(*F*^2^) = 0.141	H-atom parameters constrained
*S* = 1.00	*w* = 1/[σ^2^(*F*_o_^2^) + (0.0748*P*)^2^ + 0.3882*P*] where *P* = (*F*_o_^2^ + 2*F*_c_^2^)/3
3724 reflections	(Δ/σ)_max_ < 0.001
190 parameters	Δρ_max_ = 0.28 e Å^−^^3^
0 restraints	Δρ_min_ = −0.21 e Å^−^^3^

### Special details

**Table:**

Geometry. All e.s.d.'s (except the e.s.d. in the dihedral angle between two l.s. planes) are estimated using the full covariance matrix. The cell e.s.d.'s are taken into account individually in the estimation of e.s.d.'s in distances, angles and torsion angles; correlations between e.s.d.'s in cell parameters are only used when they are defined by crystal symmetry. An approximate (isotropic) treatment of cell e.s.d.'s is used for estimating e.s.d.'s involving l.s. planes.
Refinement. Refinement of *F*^2^ against ALL reflections. The weighted *R*-factor *wR* and goodness of fit *S* are based on *F*^2^, conventional *R*-factors *R* are based on *F*, with *F* set to zero for negative *F*^2^. The threshold expression of *F*^2^ > σ(*F*^2^) is used only for calculating *R*-factors(gt) *etc*. and is not relevant to the choice of reflections for refinement. *R*-factors based on *F*^2^ are statistically about twice as large as those based on *F*, and *R*-factors based on ALL data will be even larger.

### Fractional atomic coordinates and isotropic or equivalent isotropic displacement parameters (Å^2^)

**Table:**

	*x*	*y*	*z*	*U*_iso_*/*U*_eq_	
N1	0.01908 (8)	1.0929 (3)	0.62899 (8)	0.0564 (4)	
O1	0.03792 (7)	0.9296 (3)	0.69321 (7)	0.0604 (3)	
C1	0.11922 (9)	0.8524 (3)	0.70211 (9)	0.0487 (4)	
C1A	0.15432 (8)	0.9589 (3)	0.64670 (8)	0.0396 (3)	
C2	0.24052 (8)	0.9339 (2)	0.62856 (7)	0.0345 (3)	
H2A	0.2427	0.7768	0.6030	0.041*	
C3	0.25009 (8)	1.1335 (2)	0.56826 (8)	0.0359 (3)	
H3A	0.2545	1.2872	0.5970	0.043*	
C4	0.16899 (9)	1.1510 (2)	0.49910 (8)	0.0385 (3)	
O3	0.14932 (6)	0.90993 (17)	0.47197 (6)	0.0418 (2)	
H3B	0.1068	0.9106	0.4352	0.063*	
C5	0.09667 (9)	1.2536 (3)	0.53313 (9)	0.0467 (3)	
H5A	0.1089	1.4201	0.5492	0.056*	
H5B	0.0436	1.2497	0.4928	0.056*	
C5A	0.08853 (9)	1.1061 (3)	0.60280 (8)	0.0431 (3)	
C6	0.14961 (12)	0.6749 (4)	0.76606 (11)	0.0655 (5)	
H6A	0.1053	0.6434	0.7933	0.098*	
H6B	0.1986	0.7384	0.8030	0.098*	
H6C	0.1647	0.5275	0.7435	0.098*	
C7	0.31251 (8)	0.9425 (2)	0.70323 (8)	0.0365 (3)	
C8	0.31689 (10)	1.1287 (3)	0.75728 (9)	0.0529 (4)	
H8A	0.2767	1.2521	0.7468	0.063*	
C9	0.38009 (11)	1.1345 (4)	0.82687 (10)	0.0621 (5)	
H9A	0.3818	1.2600	0.8630	0.075*	
C10	0.44022 (10)	0.9549 (4)	0.84244 (10)	0.0601 (5)	
H10A	0.4823	0.9574	0.8894	0.072*	
C11	0.43813 (10)	0.7719 (4)	0.78874 (11)	0.0610 (5)	
H11A	0.4796	0.6520	0.7987	0.073*	
C12	0.37398 (9)	0.7651 (3)	0.71932 (9)	0.0473 (3)	
H12A	0.3726	0.6394	0.6834	0.057*	
C13	0.33153 (9)	1.1072 (3)	0.53928 (8)	0.0445 (3)	
O2	0.37936 (8)	1.2779 (3)	0.54585 (9)	0.0725 (4)	
C14	0.35025 (12)	0.8785 (4)	0.50168 (12)	0.0625 (5)	
H14A	0.4038	0.8928	0.4870	0.094*	
H14B	0.3060	0.8472	0.4551	0.094*	
H14C	0.3530	0.7477	0.5387	0.094*	
C15	0.18200 (11)	1.3114 (3)	0.43155 (9)	0.0532 (4)	
H15A	0.1305	1.3163	0.3905	0.080*	
H15B	0.2273	1.2474	0.4103	0.080*	
H15C	0.1964	1.4718	0.4512	0.080*	

### Atomic displacement parameters (Å^2^)

**Table:**

	*U*^11^	*U*^22^	*U*^33^	*U*^12^	*U*^13^	*U*^23^
N1	0.0351 (6)	0.0810 (10)	0.0512 (7)	0.0097 (6)	0.0059 (5)	−0.0093 (7)
O1	0.0367 (6)	0.0925 (9)	0.0544 (6)	0.0057 (6)	0.0159 (5)	0.0004 (6)
C1	0.0355 (7)	0.0662 (10)	0.0456 (8)	0.0015 (7)	0.0119 (6)	−0.0035 (7)
C1A	0.0320 (6)	0.0459 (7)	0.0394 (6)	0.0020 (5)	0.0050 (5)	−0.0050 (6)
C2	0.0307 (6)	0.0357 (6)	0.0359 (6)	0.0021 (5)	0.0054 (5)	−0.0022 (5)
C3	0.0344 (6)	0.0332 (6)	0.0373 (6)	−0.0010 (5)	0.0025 (5)	−0.0031 (5)
C4	0.0373 (6)	0.0355 (7)	0.0386 (6)	0.0015 (5)	0.0005 (5)	−0.0021 (5)
O3	0.0384 (5)	0.0391 (5)	0.0426 (5)	0.0001 (4)	−0.0015 (4)	−0.0072 (4)
C5	0.0412 (7)	0.0453 (8)	0.0479 (8)	0.0106 (6)	−0.0014 (6)	−0.0046 (6)
C5A	0.0326 (6)	0.0512 (8)	0.0422 (7)	0.0062 (6)	0.0018 (5)	−0.0107 (6)
C6	0.0571 (10)	0.0857 (13)	0.0595 (10)	0.0068 (9)	0.0253 (8)	0.0179 (9)
C7	0.0293 (6)	0.0426 (7)	0.0375 (6)	0.0024 (5)	0.0072 (5)	0.0026 (5)
C8	0.0445 (8)	0.0609 (10)	0.0484 (8)	0.0134 (7)	0.0008 (6)	−0.0109 (7)
C9	0.0518 (9)	0.0840 (13)	0.0460 (8)	0.0013 (9)	0.0019 (7)	−0.0165 (8)
C10	0.0404 (8)	0.0890 (13)	0.0451 (8)	−0.0007 (8)	−0.0016 (6)	0.0098 (9)
C11	0.0415 (8)	0.0714 (11)	0.0644 (10)	0.0161 (8)	0.0010 (7)	0.0143 (9)
C12	0.0398 (7)	0.0487 (8)	0.0516 (8)	0.0091 (6)	0.0073 (6)	0.0031 (7)
C13	0.0356 (7)	0.0531 (8)	0.0416 (7)	−0.0058 (6)	0.0028 (5)	0.0071 (6)
O2	0.0510 (7)	0.0752 (9)	0.0902 (9)	−0.0263 (6)	0.0141 (6)	−0.0017 (7)
C14	0.0567 (10)	0.0662 (11)	0.0734 (11)	0.0039 (8)	0.0327 (9)	0.0002 (9)
C15	0.0560 (9)	0.0499 (9)	0.0483 (8)	−0.0014 (7)	0.0013 (7)	0.0101 (7)

### Geometric parameters (Å, °)

**Table:**

N1—C5A	1.306 (2)	C6—H6B	0.9600
N1—O1	1.4090 (19)	C6—H6C	0.9600
O1—C1	1.3550 (18)	C7—C8	1.382 (2)
C1—C1A	1.357 (2)	C7—C12	1.3779 (19)
C1—C6	1.475 (2)	C8—C9	1.385 (2)
C1A—C5A	1.4117 (19)	C8—H8A	0.9300
C1A—C2	1.5032 (18)	C9—C10	1.372 (3)
C2—C7	1.5236 (17)	C9—H9A	0.9300
C2—C3	1.5514 (18)	C10—C11	1.370 (3)
C2—H2A	0.9800	C10—H10A	0.9300
C3—C13	1.520 (2)	C11—C12	1.391 (2)
C3—C4	1.5572 (17)	C11—H11A	0.9300
C3—H3A	0.9800	C12—H12A	0.9300
C4—O3	1.4257 (16)	C13—O2	1.2089 (19)
C4—C15	1.520 (2)	C13—C14	1.486 (2)
C4—C5	1.534 (2)	C14—H14A	0.9600
O3—H3B	0.8200	C14—H14B	0.9600
C5—C5A	1.487 (2)	C14—H14C	0.9600
C5—H5A	0.9700	C15—H15A	0.9600
C5—H5B	0.9700	C15—H15B	0.9600
C6—H6A	0.9600	C15—H15C	0.9600
			
C5A—N1—O1	105.32 (12)	H6A—C6—H6B	109.5
C1—O1—N1	108.49 (12)	C1—C6—H6C	109.5
O1—C1—C1A	109.66 (14)	H6A—C6—H6C	109.5
O1—C1—C6	116.05 (14)	H6B—C6—H6C	109.5
C1A—C1—C6	134.27 (14)	C8—C7—C12	118.30 (13)
C1—C1A—C5A	104.14 (13)	C8—C7—C2	120.33 (12)
C1—C1A—C2	131.88 (13)	C12—C7—C2	121.36 (12)
C5A—C1A—C2	123.94 (13)	C7—C8—C9	121.07 (15)
C1A—C2—C7	112.40 (11)	C7—C8—H8A	119.5
C1A—C2—C3	108.51 (10)	C9—C8—H8A	119.5
C7—C2—C3	111.81 (10)	C8—C9—C10	119.88 (17)
C1A—C2—H2A	108.0	C8—C9—H9A	120.1
C7—C2—H2A	108.0	C10—C9—H9A	120.1
C3—C2—H2A	108.0	C11—C10—C9	119.92 (14)
C13—C3—C2	112.53 (11)	C11—C10—H10A	120.0
C13—C3—C4	112.97 (11)	C9—C10—H10A	120.0
C2—C3—C4	111.28 (10)	C10—C11—C12	120.04 (15)
C13—C3—H3A	106.5	C10—C11—H11A	120.0
C2—C3—H3A	106.5	C12—C11—H11A	120.0
C4—C3—H3A	106.5	C7—C12—C11	120.76 (15)
O3—C4—C15	110.73 (12)	C7—C12—H12A	119.6
O3—C4—C5	109.96 (12)	C11—C12—H12A	119.6
C15—C4—C5	109.44 (12)	O2—C13—C14	121.07 (15)
O3—C4—C3	106.10 (10)	O2—C13—C3	118.51 (15)
C15—C4—C3	112.61 (12)	C14—C13—C3	120.40 (13)
C5—C4—C3	107.92 (11)	C13—C14—H14A	109.5
C4—O3—H3B	109.5	C13—C14—H14B	109.5
C5A—C5—C4	109.10 (11)	H14A—C14—H14B	109.5
C5A—C5—H5A	109.9	C13—C14—H14C	109.5
C4—C5—H5A	109.9	H14A—C14—H14C	109.5
C5A—C5—H5B	109.9	H14B—C14—H14C	109.5
C4—C5—H5B	109.9	C4—C15—H15A	109.5
H5A—C5—H5B	108.3	C4—C15—H15B	109.5
N1—C5A—C1A	112.39 (14)	H15A—C15—H15B	109.5
N1—C5A—C5	123.88 (13)	C4—C15—H15C	109.5
C1A—C5A—C5	123.71 (13)	H15A—C15—H15C	109.5
C1—C6—H6A	109.5	H15B—C15—H15C	109.5
C1—C6—H6B	109.5		
			
C5A—N1—O1—C1	−0.02 (17)	O1—N1—C5A—C1A	−0.19 (17)
N1—O1—C1—C1A	0.22 (18)	O1—N1—C5A—C5	178.08 (13)
N1—O1—C1—C6	−178.75 (15)	C1—C1A—C5A—N1	0.32 (17)
O1—C1—C1A—C5A	−0.32 (17)	C2—C1A—C5A—N1	178.46 (13)
C6—C1—C1A—C5A	178.4 (2)	C1—C1A—C5A—C5	−177.95 (14)
O1—C1—C1A—C2	−178.25 (14)	C2—C1A—C5A—C5	0.2 (2)
C6—C1—C1A—C2	0.5 (3)	C4—C5—C5A—N1	−157.04 (14)
C1—C1A—C2—C7	−46.1 (2)	C4—C5—C5A—C1A	21.04 (19)
C5A—C1A—C2—C7	136.34 (14)	C1A—C2—C7—C8	−51.35 (18)
C1—C1A—C2—C3	−170.25 (15)	C3—C2—C7—C8	70.98 (16)
C5A—C1A—C2—C3	12.17 (18)	C1A—C2—C7—C12	127.79 (14)
C1A—C2—C3—C13	−173.88 (11)	C3—C2—C7—C12	−109.88 (15)
C7—C2—C3—C13	61.59 (14)	C12—C7—C8—C9	−1.6 (2)
C1A—C2—C3—C4	−45.93 (14)	C2—C7—C8—C9	177.60 (16)
C7—C2—C3—C4	−170.45 (10)	C7—C8—C9—C10	0.8 (3)
C13—C3—C4—O3	79.39 (14)	C8—C9—C10—C11	0.7 (3)
C2—C3—C4—O3	−48.32 (14)	C9—C10—C11—C12	−1.5 (3)
C13—C3—C4—C15	−41.88 (16)	C8—C7—C12—C11	0.8 (2)
C2—C3—C4—C15	−169.60 (12)	C2—C7—C12—C11	−178.33 (14)
C13—C3—C4—C5	−162.78 (12)	C10—C11—C12—C7	0.7 (3)
C2—C3—C4—C5	69.50 (14)	C2—C3—C13—O2	−125.57 (14)
O3—C4—C5—C5A	62.22 (14)	C4—C3—C13—O2	107.38 (16)
C15—C4—C5—C5A	−175.95 (12)	C2—C3—C13—C14	56.16 (17)
C3—C4—C5—C5A	−53.09 (15)	C4—C3—C13—C14	−70.90 (17)

### Hydrogen-bond geometry (Å, °)

**Table:**

*D*—H···*A*	*D*—H	H···*A*	*D*···*A*	*D*—H···*A*
O3—H3B···N1^i^	0.82	2.08	2.8689 (17)	162

Symmetry codes: (i) −*x*, −*y*+2, −*z*+1.
